# StructRMDB: A database of RNA modification sites that affect RNA secondary structure

**DOI:** 10.1016/j.csbj.2025.11.058

**Published:** 2025-12-02

**Authors:** Ziyan Zhang, Xuan Wang, Jingxian Zhou, Jia Meng, Bowen Song, Yuxin Liang, Yuheng Cai, Jiongming Ma

**Affiliations:** a Department of Biosciences and Bioinformatics, Xi’an Jiaotong-Liverpool University, Suzhou, Jiangsu 215123, China; b Institute of Systems, Molecular and Integrative Biology, University of Liverpool, Liverpool L69 7ZB, UK; c AI University Research Centre, Xi’an Jiaotong-Liverpool University, Suzhou, Jiangsu 215123, China; d Key Laboratory of Ministry of Education for Gastrointestinal Cancer, School of Basic Medical Sciences, Fujian Medical University, Fuzhou, Fujian 350004, China; e Computational Biology Department, School of Computer Science, Carnegie Mellon University, Pittsburgh, PA 15123, USA; f Institute of Infection, Veterinary & Ecological Sciences Immunology & Vaccinology (INPR), University of Liverpool, Liverpool L69 7ZB, UK; g Sino-French Hoffmann Institute, School of Basic Medical Sciences, Guangzhou Medical University, Guangzhou, Guangdong 511436, China; h School of AI and Advanced Computing, AI University Research Centre, Xi’an Jiaotong-Liverpool University, Suzhou, Jiangsu 215123, China; i Department of Computer Science, University of Liverpool, Liverpool L69 7ZB, United Kingdom; j Department of Public Health, School of Medicine, Nanjing University of Chinese Medicine, Nanjing 210023, China

**Keywords:** RNA modification, RNA secondary structure, N6-methyladenosine, Pseudouridine, Adenosine-to-inosine editing

## Abstract

Post-transcriptional RNA modifications, prevalent in multiple RNA species such as mRNA, rRNA, and tRNA, play a significant role in biological processes by altering RNA structures. With recent advancements in prediction algorithms, it is possible to predict RNA secondary structure for sequences containing modified bases. In this study, we introduce StructRMDB, the first database designed to characterize the impact of chemical modifications on RNA secondary structure. StructRMDB comprises more than 880,000 RNA modification sites and their structural impacts, including *N*^*6*^-Methyladenosine (m^6^A), pseudouridine (Ψ), and adenosine-to-inosine editing (A-to-I) from nine species in both pre-RNA and mature RNA. Two RNA secondary structure prediction tools (RNAstructure and ViennaRNA), along with four scoring methods (Similarity Score, Relative Score, Distance, and SMC Score), were adopted to assess structural changes induced by these modifications. Additionally, we visualized RNA secondary structures with and without modifications to highlight structural alterations. A user-friendly graphical interface is provided to facilitate the querying, downloading, and sharing of modified site evaluation and annotation data, offering novel insights into the effects of RNA modifications on secondary structure. StructRMDB serves as a valuable resource for studying the structural impact of RNA modifications and is available at: http://www.rnamd.org/StructRMDB/index.html.

## 1 Introduction

Single-stranded RNA molecules can fold into a wide spectrum of secondary and tertiary structures that underpin essential biological mechanisms, including catalytic ribozyme activity, temperature or metabolite sensing, and the epigenetic regulation of long non-coding RNAs 1, 2, 3, 4. RNA structural states themselves may act as heritable carriers of epigenetic information, a concept referred to as RNA structural memory [1]. Furthermore, mutations that alter the structure of RNA have been linked to human diseases such as dysduplication, retinoblastoma, and breast cancer [5]. Therefore, understanding RNA folding and structure is critical for advancing our understanding of RNA functions.

RNA secondary structure, a central component of RNA folding, is characterized by distinct features such as hairpins, long-range interactions, G-quadruplexes, R-loops, and pseudoknots. These structures arise from interactions between non-adjacent nucleotides [6] and profoundly influence key mRNA processes, including transcription, splicing, and translation [7]. The successful identification of RNA secondary structure is highly informative. For example, incorporating secondary structure information has been shown to improve triplex-forming oligonucleotide (TPX/TFO) prediction specificity for lncRNAs [8]. Additionally, transcriptional regulation including directionality, dynamics, and RNA splicing relies on RNA secondary structures, which enhance sequence complexity [9]. However, RNA secondary structures are highly dynamic and often regulated by RNA-binding proteins (RBPs), making them difficult to predict solely from primary sequences [6].

In addition to secondary structure, RNA chemical modifications also play pivotal roles in regulating RNA stability and function by altering the chemical properties of individual nucleotides [6]. These modifications can reshape RNA–protein interactions because many RBPs either directly recognize specific modifications or are sensitive to the structural changes they induce [10]. Moreover, modifications can alter secondary structure formation, ranging from slight stabilization to significant destabilization, depending on the position and sequence context of the modification. In some cases, the modification can induce substantial structural rearrangements, such as converting a hairpin to a duplex [11]. For example, m^6^A modifications destabilize RNA helices and modulate regulatory processes [12], while the positively charged *N1*-methyladenosine (m^1^A) can locally alter mRNA structure near translation initiation sites by disrupting Watson-Crick base pairing [13]. Similarly, the secondary structure can influence the modification level, as some studies suggest that folded RNA secondary structures can prevent motifs from being methylated after transcription [14].

This bidirectional relationship underscores the necessity of accurately identifying structural regions affected by chemical modifications. Such interplay has been observed in mitochondrial diseases, where tRNA mutations alter modification levels and structural stability, ultimately impairing translation [14]. Moreover, the interaction between modifications and secondary structure has been demonstrated through the analysis of RNA structuromes in HIV, yeast, Arabidopsis, and mammalian cells and tissues, highlighting the importance of RNA modifications in secondary structure, particularly in the context of human diseases [15].

Currently, the integration of modification effects into RNA structural prediction remains challenging due to the lack of specialized algorithms and precise thermodynamic parameters. The prediction of RNA secondary structure has long been a major focus in computational biology. Traditional algorithms commonly identify the structure with the minimum free energy (MFE) using thermodynamic parameters derived from experimental data [16]. Existing tools have made preliminary attempts to address this issue. For example, RNAstructure incorporates a nearest-neighbor model that allows folding predictions for modified nucleotides, such as m^6^A, by extending thermodynamic parameters [17]. In contrast, ViennaRNA adopts a flexible constraint framework that dynamically adjusts base-pairing probabilities to reflect the energetic effects of modifications like m6A, inosine, and pseudouridine [18].

Given the regulatory significance and widespread occurrence of RNA modifications [19], structure prediction based on modifications can help researchers locate modification sites that may influence RNA structure and interpret potential indirect functional effects mediated by these modifications. To address this need, we developed StructRMDB, a predicted structure-centric database dedicated to screening and identifying RNA secondary structures altered by single-site RNA modifications, including m⁶A, pseudouridine (Ψ), and A-to-I RNA editing.

**Fig. 1:**
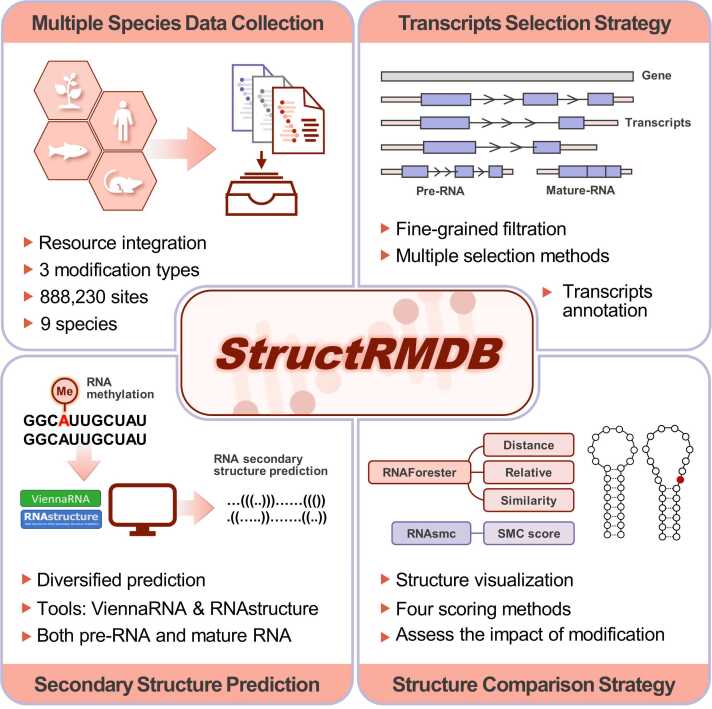
Layout of StructRMDB database. StructRMDB is a comprehensive database that focuses on RNA secondary structure changes influenced by single-base modifications, including m^6^A, pseudouridine (Ψ), and RNA-editing (A-to-I). Both ViennaRNA and RNAstructure were applied to predict RNA secondary structures with and without modifications, and four scores were used to quantify the resulting structural alterations. In addition, the database provides extensive functional annotations and visualization of RNA secondary structures.

In contrast to other RNA structural databases, such as the Nucleic Acid Circular Dichroism Database —a repository of experimentally derived circular dichroism spectra of nucleic acids [17], StructRMDB is dedicated to exploring the potential effects of chemical modifications on RNA secondary structures through large-scale predictive data. It integrates advanced structure prediction algorithms with more than 880,000 high-confidence modification sites across nine species, enabling direct comparisons between modified and unmodified RNA structural states. Furthermore, the database offers comprehensive functional annotations, including gene regions, gene types, RNA sequence categories (pre-RNA and mature RNA), as well as overlaps with RNA-binding proteins (RBPs), miRNAs, and single nucleotide polymorphisms (SNPs) associated with modification sites. RNAplot was used to visually display predicted RNA secondary structures with or without modifications (Fig. 1). StructRMDB is now freely accessible at: http://www.rnamd.org/StructRMDB/index.html.

## 2 Material and methods

### 2.1 Workflow

**Fig. 2:**
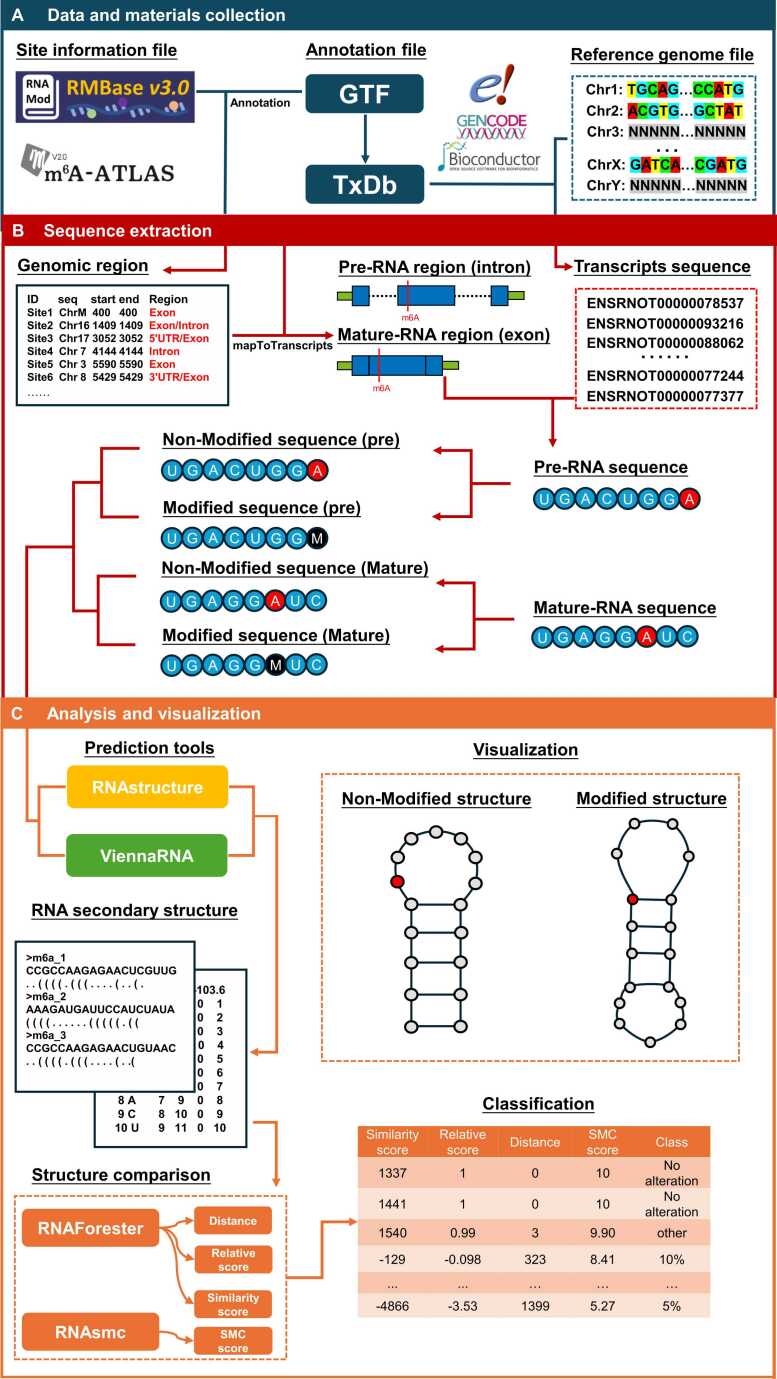
Workflow of data processing. The process involves three parts: data and materials collection, sequence extraction, and analysis and visualization. (A) Data and materials collection. Modification information, reference files, and annotation files were sourced from public databases, including RMBase V3.0, m^6^A-Atlas V2.0, GENCODE, Ensembl, and UCSC Genome Browser. (B) Sequence extraction. Genomic regions were annotated and mapped to transcripts to generate mature and pre-RNA sequences containing the modified sites. For each modification type (e.g., m⁶A, Ψ, A-to-I), sequences were generated in two versions: one containing the original base and another incorporating the specific modified base symbol. (C) Analysis and visualization. RNA sequences were analysed by using RNAstructure and ViennaRNA, and the results were formatted in Connectivity Table (CT) and dot-bracket notation. Predicted structures of modified and unmodified RNA were compared using four indices, classified into categories, and visualized through secondary structure plots.

The workflow of StructRMDB involves three main steps: data and materials collection, sequence extraction, analysis and visualization (Fig. 2). Information on modification sites was collected from RMBase V3.0 and m^6^A-Atlas V2.0, along with annotation and reference genome files from GENCODE 2021, UCSC Genome Browser, and Ensembl. During sequence extraction, genomic regions for each site were annotated. By integrating these annotations with transcript sequences, both mature and pre-RNA sequences containing the modified sites were generated. Additionally, the RNA sequences were analysed using the prediction tools RNAstructure V6.4 and ViennaRNA 2.6.4. Results from these two software were formatted in either Connectivity Table (CT) or dot-bracket notation. The predicted structures for both modified and unmodified RNA were compared using four indices to assess their influence, and the results were classified into four categories. Finally, the predicted secondary structure of modified and unmodified RNA was visualized through secondary structure plots.

### 2.2 Data and material collection

Information on three types of chemical modification sites across nine species was collected to assess their impact on secondary structure. Data on m^6^A-modified RNA sites were collected from the database m^6^A-Atlas V2.0 [20], while information on pseudouridine and adenosine-to-inosine was obtained from the database RMBase v3.0 [21]. The annotation files for each species were downloaded from GENCODE 2021[22] and Ensembl [23]. The reference genome sequences were mainly downloaded from Ensembl, with some data obtained from GENCODE 2021 and UCSC Genome Browser (Supplementary Table 1). Next, the downloaded GTF files were used to annotate each modification site, identify its genomic region, and determine the corresponding RNA sequence type. If a modified site is annotated as intergenic, no RNA sequence will be generated. If the annotated genomic region comprises introns without exons, this site will only yield a pre-RNA sequence. Similarly, the site will yield a mature RNA sequence if its annotated genomic region contains exons. In this database, both pre-RNA and mature RNA sequences were considered, meaning one modified site could have both sequence types.

### 2.3 Sequence extraction

The genomic coordinates of transcripts in the GTF file were extracted using the R package GenomicFeatures [24] and subsequently used to retrieve transcript sequences from the reference genome. Following acquisition of transcriptome-wide modification sites and corresponding sequences, a 401 bp sequence (comprising 200 bp upstream, 200 bp downstream, and the modification site) was extracted for secondary structure prediction. In cases where modification sites were mapped to multiple transcript isoforms, the sequence from the longest transcript was preferentially selected. When the required 401 bp sequence could not be obtained, either due to proximity to transcript termini or when the total transcript length was insufficient (Supplementary Figure 1 A), the maximum available sequence length was utilized instead. In some cases, this occurred when the sites were located within shorter non-coding RNAs such as tRNAs and rRNA.

For mature RNA sequence extraction, the R packages GenomicFeatures [24], BSgenome [25], and Biostrings [25] in Bioconductor [25] were used to construct the regions of mature RNA. Furthermore, the corresponding exon information was extracted and merged based on the species-specific BSgenome object to construct the mature RNA sequence. Similar to the pre-RNA sequence extraction, the longest sequence was prioritized for analysis (Supplementary Figure 1B). In this way, each modification site in the genome could have up to two RNA sequences (pre- and mature), each with a corresponding transcriptome name. A specific symbol was added at the modification site in the modified sequences to differentiate them from the original, unmodified sequences.

### 2.4 Analysis and visualization

#### 2.4.1 Prediction and comparison of secondary structure

The extracted sequences were subjected to secondary structure prediction using RNAstructure 6.4 [26] and ViennaRNA 2.6.4 [27]. The ViennaRNA tool is capable of predicting secondary structures containing three types of RNA modifications: m6A, pseudouridine, and adenosine-to-inosine. In contrast, RNAstructure can only predict secondary structures with m6A modifications. To compare the differences between modified and unmodified secondary structures, the predicted secondary structures, provided in the form of dot-bracket files or Connectivity Tables (CT), were input into RNAforester 2.0.1 [27] and RNAsmc 0.8.0 [28] for evaluation. A total of four types of scores were employed for this evaluation: three from RNAforester—namely “similarity”, “relative similarity”, and “distance”—and one from RNAsmc, the SMC score.

### 2.5 RNAforester

RNAforester accepts the primary structure of RNA and its secondary structure in the form of a dot-bracket notation, calculates RNA secondary structure alignments, and performs the comparison based on the tree alignment model. The similarity score and distance were provided by RNAforester through a global alignment of RNA secondary structures. The scoring methods are summarized in Table 1.

**Table 1:** The scoring method of global similarity and distance (Indel = insertion–deletion).

Scoring type	Global similarity	Distance
Pair match	10	0
Pair indel*	-5	3
Base match	1	0
Base replacement	0	1
Base indel	-10	2

The relative similarity score is derived from the similarity score, but it undergoes a normalized process as per the following equation:

$$\mathrm{RS}(a,b)=2\times \frac{S(a,b)}{S(a,a)+S(b,b)}$$

Where a,b represents the secondary structure of two sequences. RS represents for Relative Similarity and S represents for Similarity.

At this stage, the relative similarity score is capped at 1, effectively eliminating the influence of the length of the secondary structure. The distance score, another evaluation metric provided by RNAforester, increases with the disparity between two structures, contrasting with the similarity score. When two structures are identical, the distance score is 0. It should be noted that the distance score is also influenced by the sequence length. Only the optimal alignment score is taken into consideration.

### 2.6 RNAsmc

RNAsmc implements a strategy for dynamic alignment based on structural motifs. It accepts the Connectivity Table (CT) file as input, identifying and annotating structural motifs within the RNA. The output includes a score ranging from 0 to 10, where a score of 10 indicates no structural changes. Notably, RNAsmc demonstrates strong robustness to variations in sequence length [28].

$$\mathrm{SMCscore}=\frac{5}{6}\times [\sum_{U\in \left\{B,E,H,I,M,S\right\}}\frac{\left|U_{p1}\cap U_{p2}\right|}{\left|U_{p1}\cup U_{p2}\right|}\;+\;\sum_{U\in \{B,E,H,I,M,S\}}\frac{\min(U_{n1},U_{n2})}{\max(U_{n1},U_{n2})}]$$

Where B,E,H,I,M, and S represent as the bulge loop, external loop, hairpin loop, interior loop, multiple branch loop, and stem, correspondingly. $U_{p1}$ and $U_{p2}$ represent the spatial arrangement sets of motifs within RNA1 and RNA2 for each type of motif, respectively. $U_{n1}$ and $U_{n2}$ represent the quantities of motifs in these two RNAs. The first item represents the Jaccard similarity coefficient, and the secondary item represents the likelihood ratio.

After comparison, we will perform visualization of RNA secondary structure before and after modification using RNAplot [29], with the modified position highlighted in the plot.

### 2.7 RNA secondary structure modification classification

Currently, the study of RNA secondary structures is still in the developmental stage. It remains computationally challenging to accurately determine whether differences in RNA structures can affect their functions. To assist users in identifying RNA structures with significant differences, this study employs the Relative, Distance, and SMC scores for classification. The global similarity score is omitted because it varies with sequence length and is therefore not robust in this scenario. Specifically, sequences with no structural differences (Relative score = 1, Distance = 0, and SMC score = 10) are classified as “No alteration”. For sequences with differences, the three scores are used for ranking. For example, a sequence is classified in the “Top 10 %” category only if all three scores fall within the top 10 % of severe structural changes in the dataset. (The Relative score and SMC score are ranked in ascending order, while the Distance score is ranked in descending order, and the top 10 % from each ranking is selected). Similarly, sequences where all three scores fall between 10 % and 50 % are classified into the “10 %-50 %” category, and those with scores between 50 % and 99.9 % are classified in the “50 %-99.9 %” category (Table 2).

**Table 2:** Classification rules for modified RNA secondary structures.

Category	Criteria	Structural Interpretation
No alteration	- Structural distance = 0 - Relative score = 1.0 - RNA-SMC score = 10	No change in RNA secondary structures
50 %-99.9 %	Site ranks in top 50 %-99.9 % for: - Relative score - Structural distance - RNA-SMC score	Modest alterations in RNA secondary structure
10 %-50 %	Site ranks in 10 %-50 % for all: - Relative score - Structural distance - RNA-SMC score	substantial but minor structural changes
10 %	Site ranks in top 10 % for all: - Relative score - Structural distance - RNA-SMC score	the most significant structural alterations

### 2.8 Molecular interaction annotation

In addition, StructRMDB provides RNA-binding proteins (RBPs), microRNAs (miRNAs), and single nucleotide polymorphisms (SNPs). These could help to investigate the connections to other epi-transcriptomic markers and the roles of modifications in gene expression regulation and disease development. The RBPs-binding sites were acquired from POSTAR3 [30]. The miRNAs target sites were retrieved from starBase v2.0 [31]. The SNP information was annotated by Ensembl [23].

### 2.9 Database construction

The MySQL Database Management System was applied to store and manage all datasets in StructRMDB. Hypertext Preprocessor (PHP) and JavaScript were used to develop the database queries and user interface. The layout and rendering of the web interface were built using HyperText Markup Language (HTML) and Cascading Style Sheets (CSS). Query results can be visualized in various statistical graph forms using DataTables, ECharts, and HighCharts. JBrowse was implemented to navigate all genomic tracks on the web server.

## 3 Results

StructRMDB, equipped with a user-friendly web interface, facilitates the comprehensive exploration of chemical modifications, RNA secondary structures, and corresponding annotations. The platform offers multiple options for filtering and selecting modification sites, allowing users to tailor their analyses. For instance, users can select results from RNAstructure or ViennaRNA and subsequently choose detailed options for further analysis.

### 3.1 StructRMDB assesses structural changes induced by several RNA modifications

A total of 791,825 m^6^A modification sites were collected from m^6^A-Atlas V2.0. After processing, approximately 1446,500 unique result IDs were generated in StructRMDB using RNAstructure and ViennaRNA separately. Among these, around 740,000 result IDs pertain to pre-RNA, and around 700,000 result IDs pertain to mature-RNA. Detailed information is shown in Table 3. Results from A-to-I and pseudo-modifications were given by ViennaRNA only, including both pre-RNA and mature RNA results. Detailed information is provided in Table 4. Annotation information, including RBPs, miRNAs, and SNPs, is shown in Supplementary Table 2.

**Table 3:** RNA m^6^A modification statistics across in m^6^A-Atlas V2.0.

Species	Assembly	Site number	Pre-RNA		Mature-RNA	
			**RNAstructure**	**ViennaRNA**	**RNAstructure**	**ViennaRNA**
Homo sapiens	hg38	422,730	394,631	394,594	363,242	363,212
Mus musculus	mm10	266,632	261,142	261,142	252,205	252,193
Rattus norvegicus	Rn6	6144	5470	5469	5378	5377
Arabidopsis thaliana	TAIR10	35,329	24,204	24,203	23,781	23,780
Drosophila melanogaster	BDGP6	25,570	25,335	25,333	24,588	24,582
Saccharomyces cerevisiae	sacCer3	10,560	10,555	10,553	10,520	10,518
Danio rerio	GRCz10	24,860	23,108	23,089	22,275	22,332

**Table 4:** Other two modification statistics across in RMBase, predicted by ViennaRNA only.

Species	Assembly	Modification	Site number	Pre-RNA	Matrue-RNA
Homo sapiens	hg38	A-to-I	142,648	127,985	29,854
	hg38	pseudo	4835	3868	3162
Mus musculus	mm10	A-to-I	6144	7401	2486
	mm10	pseudo	35,329	3528	3354
Rattus norvegicus	rn6	A-to-I	10	4	4
	rn6	pseudo	1317	897	861
Bos taurus	bosTau9	pseudo	381	68	5
Oryctolagus cuniculus	OryCun2	pseudo	225	2	0

**Fig. 3:**
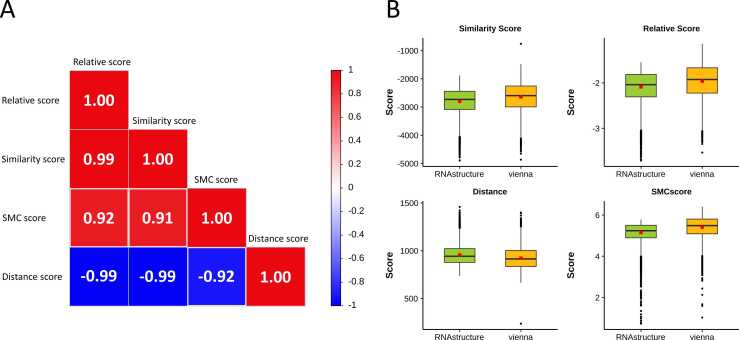
Comparison of RNA secondary structure based on RNAforester and RNAsmc. (A) Heatmap showing the Pearson correlation coefficients among four structure-comparison scores: similarity, relative, distance, and SMC scores. The coefficients quantify the degree of linear correlation between each pair of metrics, where values close to 1 or –1 indicate strong positive or negative relationships, respectively. (B) Boxplots showing the distribution of the four scores within the “10 % alteration” structural category, computed separately using RNAstructure and ViennaRNA predictions.

We performed a correlation analysis on four scores utilizing the entire dataset (Fig. 3A). The absolute values of the correlation coefficients between each pair of scores were above 0.9, indicating a strong relationship among these scores. This result demonstrated that classification based on the combination of these scores effectively reflects the status of the RNA secondary structure. As shown in Fig. 3B, the score distribution within the “10 %” category highlights notable points that may lead to significant structural changes. Under this method, the classification results of RNAstructure and ViennaRNA showed a narrower gap.

#### 3.1.1 Case study on gene METTL3

**Fig. 4:**
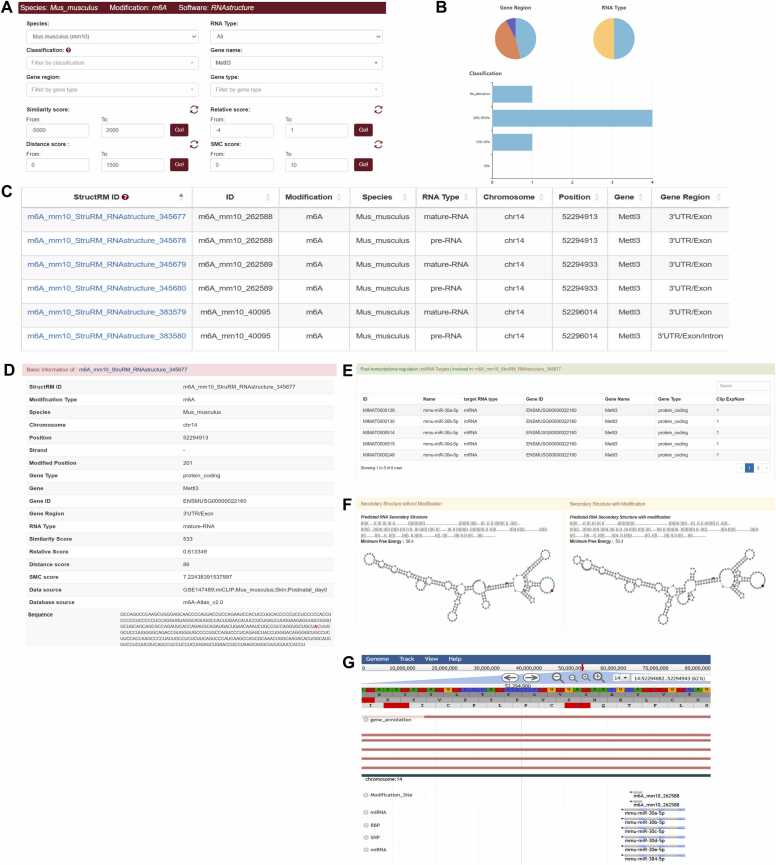
The m^6^A site of gene Mettl3 and related information in StructRMDB. (A) Filtration options of the modification site. (B) The statistical graphs returned by the chosen setting. (C)The basic information of the filtered site. (D) The details about the site include extra information about the primary structure, secondary structure, Data source (E) Detailed RBPs and miRNAs, and SNPs information associated with the modification site. Here are the miRNAs of a specific site m6A_mm10_StruRM_RNAstructure_345677. (F) Visualization of a specific site m6A_mm10_StruRM_RNAstructure_345677. (G) JBrowse of a specific site m6A_mm10_StruRM_RNAstructure_345677(m6A_mm10_262588).

Methyltransferase-like 3 (METTL3) is the most well-known m^6^A methyltransferase, playing a crucial role in the reversible epi-transcriptomic regulation of m^6^A modification [32]. A previous study indicated that m^6^A can affect RNA secondary structure in METTL3-knockout cells. This effect may result from the structural selectivity of the m^6^A modification machinery for unpaired bases [33]. In StructRMDB, you can search for "METTL3" in the "Gene name" search box after selecting a prediction tool, such as RNAstructure, and the species "Mus musculus". Different filtration options are available as well on the webpage, allowing users to quickly filter the results that meet the requirements (Fig. 4A). According to your selection, the webpage will generate statistical graphs and return the filtered modification sites (Fig. 4B and Fig. 4C). Click a specific site ID (e.g., m6A_mm10_StruRM_RNAstructure_345677), and detailed information such as primary structure, secondary structure, and data source (Fig. 4D) is shown. Additionally, RBPs, miRNAs, and SNPs information are integrated into the website to assist in exploring the post-transcriptional machinery. For the site m6A_mm10_StruRM_RNAstructure_345677, the database indicates that it is located within the interaction range of 6 miRNAs (Fig. 4E). Visualization is displayed at the bottom of the page to help users understand the effect of this site on RNA secondary structure more intuitively (Fig. 4F). Users can also click “JBrowse” to view the modification site in the genome browser (Fig. 4G).

#### 3.1.2 Case study on MALAT1

**Fig. 5:**
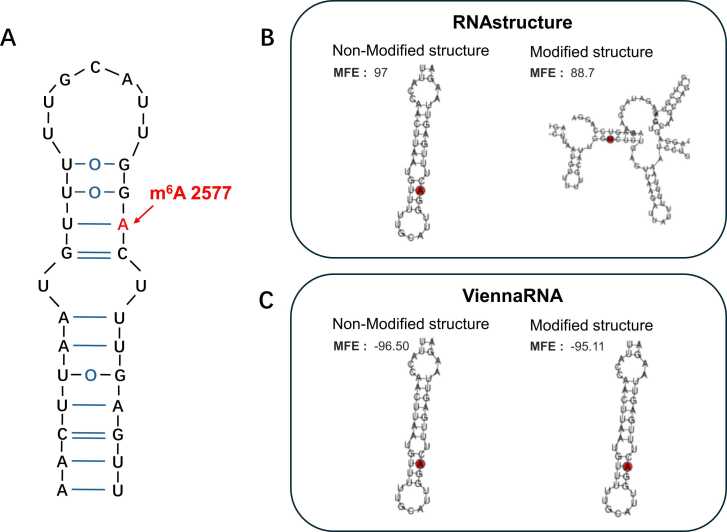
The m^6^A modification on the MALAT1 gene destabilizes its secondary RNA structure and the corresponding information in StructRMDB. (A) A validated secondary structure of the *MALAT1* hairpin, with the m^6^A site at position 2577 highlighted in red. (B) The local secondary structure and MFE values before and after the m^6^A modification, obtained using RNAstructure (m6A_hg38_StruRM_RNAstructure_692104). (C) The local secondary structure and MFE values before and after the m⁶A modification, obtained using ViennaRNA (m6A_hg38_StruRM_vienna_692047). Since the pre-RNA and mature RNA share the same sequence at this site, only one is shown.

A m^6^A site located within the hairpin stem of the human long noncoding RNA Metastasis Associated Lung Adenocarcinoma Transcript 1 (MALAT1) was identified by Liu et al. [12]. In StructRMDB, this site corresponds to m6A_hg38_StruRM_RNAstructure_692104 (RNAstructure) and m6A_hg38_StruRM_vienna_692047 (ViennaRNA). As shown in Fig. 5A, the m^6^A modification occurs at position 2577 and is highlighted in red. Both of prediction programs accurately reproduced the hairpin stem structure in the original, unmodified sequence (Figs. 5B and 5C). After the introduction of the m^6^A modification, the secondary structure predicted by RNAstructure changed, whereas ViennaRNA retained the original conformation. Notably, both prediction tools exhibited decreased absolute MFE values following m^6^A modification, suggesting reduced structural stability [34]. This observation is consistent with experimental findings from Liu et al. [12], who demonstrated that m^6^A residues within RNA stems can destabilize RNA duplexes. Specifically, m^6^A at position 2577 in MALAT1 tends to disrupt base stacking and pairing interactions, thereby decreasing local thermodynamic stability, an effect that aligns well with our computational predictions. In addition, HNRNPC, which recognizes m⁶A-induced RNA structural rearrangements through the “m^6^A-switch” mechanism described by Liu *et al.* [9], was identified in StructRMDB’s RBP annotation section. This allows users to conveniently access relevant information and facilitates the generation of more concrete biological insights from the data.

## 4 Discussion

The prediction of RNA secondary structure is crucial for understanding its function, as structure largely determines RNA stability, localization, and intermolecular interactions. Accurate structural modelling is also vital for computational design strategies, as demonstrated by recent work showing that modelling the target secondary structure can significantly improve nucleic acid library design and screening efficiency [35]. However, most existing predictive tools share a significant limitation: they fail to adequately account for the influence of RNA modifications on secondary structure. Against this backdrop, RNAstructure and ViennaRNA have emerged as two of the few tools capable of handling large-scale datasets while simultaneously incorporating modification-specific thermodynamic parameters. This unique combination of features establishes them as the most practical options for modification-inclusive predictions at present, despite their inherent limitations. In StructRMDB, we currently focus on the effects of three well-characterized RNA modifications, including m^6^A, pseudouridine (Ψ), and adenosine-to-inosine (A-to-I) editing, on RNA secondary structures. The fact that these modifications have been experimentally validated in previous research lends reliability and biological relevance to our predictions 17, 27.

While StructRMDB serves as a valuable platform for exploring the effects of RNA modifications on secondary structure, its current version has certain limitations in data coverage, algorithmic integration, and model complexity, which also highlight directions for future improvement. First, the scope and depth of the database can be further expanded. Future iterations could incorporate additional modification types, such as *N*^*5*^-methylcytosine (m^5^C), *N*^*7*^-methylguanosine (m^7^G), and *N*^*1*^-methyladenosine (m^1^A), as well as broaden the range of included species. Second, discrepancies between prediction tools represent an unavoidable challenge. Different software packages use distinct built-in thermodynamic parameters, which can lead to variation in predicted structures for the same modified sequence. For example, RNAstructure and ViennaRNA may yield different results depending on how each model incorporates modification-induced energy changes. At present, StructRMDB provides separate outputs for each algorithm, as reconciling these methodological differences is not yet feasible. Third, the underlying algorithmic approach for identifying modified bases remains imperfect and may result in inaccuracies. Errors may arise from limitations in the underlying thermodynamic data, particularly those derived from optical melting experiments using small synthetic oligonucleotides [36], or from insufficient experimental evidence for certain modification contexts.

At the algorithmic level, the core limitation lies in the simplified single-modification assumption and limited data coverage. The lack of experimentally determined thermodynamic parameters for different combinations of modifications restricts structural predictions to a single modification type per RNA sequence [18]. This simplified model fails to capture the synergistic or antagonistic effects of multiple co-occurring modifications, which are biologically prevalent in molecules such as tRNAs and rRNAs [37]. Meanwhile, it is also important to develop specialized algorithms for different types of RNA that account for modifications. For example, accurately predicting the secondary structure of lncRNAs remains challenging because the presence of pseudoknots remains a major obstacle [38]. Addressing these issues relies on two primary strategies: [1] The field needs innovation in predictive tools and experimental validation to expand the range of modifications that can be accurately modelled. The recent inclusion of RNA structure prediction in AlphaFold 3 [39], for instance, presents a promising avenue for future development of tools capable of predicting RNA secondary structures incorporating modifications. [2] It is crucial to integrate high-resolution datasets from technologies like nanopore sequencing and high-resolution mass spectrometry, which provide site-specific information on coexisting modifications, thereby providing the data foundation for constructing complex energy models 40, 41. Although the accuracy of nanopore sequencing requires further improvement, its unique principle—distinct from that of second-generation sequencing—holds promise for resolving challenges related to multiple coexisting modifications and transcript isoform ambiguity, thereby enhancing the accuracy of RNA structure prediction in complex modification contexts.

At the methodological level, technical challenges remain, particularly those arising from sequence length bias and the approach used for classification. Although the selection of a default 401 bp sequence length is not optimal, its prediction accuracy does not appear to decrease significantly. According to previous studies, when the sequence length is ≤ 700 bp, the average prediction accuracy of minimum free energy (MFE)-based methods can reach approximately 73 % [38]. Meanwhile, our case study on MALAT1 successfully reproduced the authentic local secondary structure as confirmed by biological experiments, even though the sequence length was not identical to the original [12]. Nevertheless, the influence of sequence length should not be overlooked. Both the overall RNA length and the position of the modified base can affect prediction outcomes at multiple stages of analysis. For instance, during parameter testing, the chemical stability of phosphodiester bonds in certain oligoribonucleotides has been shown to vary with sequence length [42], suggesting that sequence-dependent physicochemical properties may partially account for prediction variability. Currently, there is no established benchmark for defining categories of structural alteration. To address this, our classification method provides a practical way for users to differentiate modification sites that influence RNA structural changes and to efficiently identify those that may have a substantial impact. In the future, refinements could incorporate more robust methods to assess structural alterations and further refine the classification process.

In summary, the future objectives for StructRMDB are to expand its repertoire of modification models, incorporate additional modification types and species to provide broader insights into modification–structure relationships, and integrate a wider range of modification-aware tools for comprehensive structural evaluation. Improved assessment methods could also provide more scientifically grounded interpretations of structural alterations. By systematically addressing these limitations, StructRMDB is poised to become a more comprehensive and reliable resource for elucidating the regulatory roles of RNA modifications on structural dynamics, thereby facilitating the discovery of their potential biological functions.

## CRediT authorship contribution statement

**Jia Meng:** Writing – review & editing, Supervision, Resources, Project administration, Funding acquisition. **Jingxian Zhou:** Writing – review & editing. **Xuan Wang:** Visualization, Resources, Methodology. **Ziyan Zhang:** Writing – original draft, Visualization, Validation, Software, Methodology, Investigation, Formal analysis, Data curation. **Jiongming Ma:** Writing – review & editing, Validation, Supervision, Project administration, Methodology, Investigation. **Yuheng Cai:** Writing – review & editing, Supervision. **Yuxin Liang:** Visualization. **Bowen Song:** Writing – review & editing, Supervision.

## Funding

10.13039/501100001809National Natural Science Foundation of China [31671373]; XJTLU Key Program Special Fund [KSF-E-51 and KSF-P-02]. This work is Supported by the Supercomputing Platform of Xi’an Jiaotong-Liverpool University.

## Declaration of Competing Interest

The authors declare no competing interests.

## Footnotes

Appendix A Supplementary data associated with this article can be found in the online version at doi:10.1016/j.csbj.2025.11.058.
