# Supplementary material for: StructRMDB: A database of RNA modification sites that affect RNA secondary structure
Source: Comput Struct Biotechnol J. 2025 Dec 2;27:5493–502. doi: 10.1016/j.csbj.2025.11.058 (PMC12720313; doi:10.1016/j.csbj.2025.11.058)
Supplement: Supplementary file 1 — Supplementary material [file mmc1.pdf]

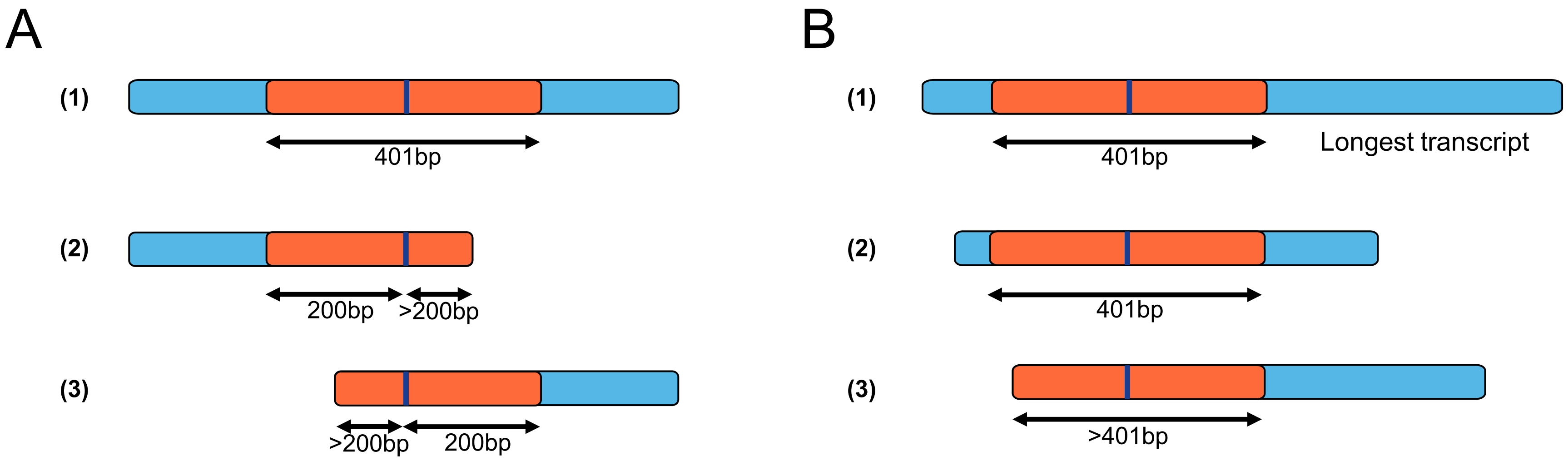

Supplementary Figure 1. Sequence extraction strategy. (A) Under normal conditions, a total of 401bp sequences were extracted from the transcriptome (1). Sequences taken from near the beginning (2) or end (3) of the transcriptome result in shorter sequences. Similarly, transcriptomes shorter than 401bp yield shorter sequences. (B) When a site maps to multiple transcriptomes, the longest sequence among them was chosen. In this scenario, either transcript (1) or transcript (2) will be chosen.
